# Case Report: Long-standing multisystem Wilson's disease in a pregnant woman with prolonged chelation discontinuation

**DOI:** 10.3389/fmed.2026.1950184

**Published:** 2026-09-17

**Authors:** Sidi Li, Xiaojie Zhang, Yanju Zhu, Yuntao Hao, Luwen Zhao

**Affiliations:** 1 The First Department of Gynecology, Affiliated Hospital of Chengde Medical University, Chengde, Hebei, China; 2 The Department of Obstetrics, Affiliated Hospital of Chengde Medical University, Chengde, Hebei, China

**Keywords:** copper metabolism disorder, multidisciplinary team consultation, periodic intensive decoppering, pregnancy, wilson's disease

## Abstract

Wilson's disease (WD) is a rare autosomal recessive copper metabolism disorder. Long-standing multisystem WD in pregnancy poses substantial maternal and fetal risks, which deteriorate markedly if copper chelation therapy is completely discontinued. We present a 37-year-old primigravida with a 23-year history of liver, brain, and kidney involvement from WD. She voluntarily discontinued all chelating agents more than a year prior to conception and received no copper-lowering treatment or routine monitoring during gestation. Her non-adherence coincided with progressive neurological impairment in the first trimester and preeclampsia in the third trimester. No multidisciplinary team (MDT) assessment was conducted during prenatal care, yet urgent MDT consultation was initiated when she was admitted at 38 + 5 weeks’ gestation with new-onset hypertension (blood pressure 141/92 mmHg), proteinuria (0.609 g/24 h), and serum ceruloplasmin of 81 mg/L. After full interdisciplinary evaluation, the MDT proposed emergency cesarean delivery. A healthy male newborn was delivered (Apgar scores 9-10-10; birth weight 3.42 kg). Despite maternal neurological deterioration and preeclampsia, critical survival of both mother and neonate was achieved through urgent intervention. This case highlights a clinical paradox in which the patient's unique history of periodic intensive decoppering might have contributed to a potential hepatic functional reserve, although this hypothesis remains speculative and unproven. The favorable outcome should not be misinterpreted as evidence that prolonged chelation interruption is tolerable; rather, it underscores the grave risks of non-adherence and the critical role of rescue MDT intervention.

## Highlights

- We describe a rare instance of full-term pregnancy in a 37-year-old woman with Wilson's disease (WD) involving the liver, brain, and kidneys for 23 years.
- Discontinuation of copper chelation therapy coincided with worsening of tremors in early pregnancy and subsequent preeclampsia.
- A multidisciplinary team (MDT) approach facilitated a safe cesarean delivery, resulting in favorable maternal and neonatal outcomes despite ongoing non-adherence to therapy.
- This case underscores the critical need for continuous maintenance therapy and strict monitoring throughout pregnancy in high-risk WD patients

## 1 Introduction

Wilson's disease (WD), an autosomal recessive copper metabolic disorder driven by biallelic ATP7B mutations, has a global prevalence of 1 in 26,000–30,000 individuals (1). Impaired biliary copper excretion triggers toxic copper deposition in the liver, brain, kidneys and cornea, manifesting as liver dysfunction, neurological and psychiatric lesions, as well as characteristic Kayser-Fleischer (K-F) rings (2). The diagnosis is established by a combination of clinical features, biochemical tests (low ceruloplasmin, elevated urinary copper), and genetic analysis (3).

WD patients face reduced fertility and increased rates of miscarriage, which are attributed to copper-mediated ovarian and endometrial damage as well as to pregnancy-induced disruption of copper homeostasis, thereby increasing the risk of acute liver failure (4, 5). Standard chelators (penicillamine, trientine) alleviate copper overload yet carry potential teratogenicity, creating a major clinical trade-off (4, 5); clinical guidelines recommend individualized dose adjustment rather than complete discontinuation of chelation therapy during pregnancy (6). However, real-world data on patient adherence to these guidelines—particularly regarding complete cessation of chelation therapy before and during pregnancy—remain extremely limited. This knowledge gap hampers the prediction of maternal and fetal outcomes in non-adherent patients and highlights the unique value of documenting such rare cases.

We report a rare case of a 37-year-old primigravida with a 23-year history of multi-organ WD (hepatic, neurological, renal) who completely discontinued chelation therapy before and during pregnancy. Despite the high baseline risks of untreated WD in pregnancy, urgent multidisciplinary team (MDT) consultation led to emergency cesarean delivery, resulting in satisfactory short-term maternal and neonatal outcomes. We caution that this favorable outcome should not be interpreted as validating treatment interruption. At initial presentation at 38 + 5 weeks of gestation, the patient presented with progressive tremors, new-onset hypertension, proteinuria, biochemical findings suggestive of copper dyshomeostasis, necessitating urgent MDT involvement.

## 2 Case presentation

### 2.1 Past medical history

A 37-year-old primigravida with a 23-year history of multisystem WD, complicated by nodular cirrhosis, extrapyramidal lesions, and chronic renal impairment manifested by persistent proteinuria. At age 14, she initially presented with upper limb tremors. Brain MRI findings suggested WD, a diagnosis subsequently confirmed by the presence of K-F rings and classic clinical manifestations. She received combination chelation therapy with penicillamine, zinc sulfate, and sodium dimercaptopropanesulfonate (DMPS). Although her symptoms improved temporarily, she experienced a severe neurological relapse six months later, manifesting as tremors, ataxia, dysarthria, and dysphagia. Intensive inpatient decoppering treatment led to significant improvement. For 21 years following her diagnosis, she adhered to a regimen of daily oral chelation and, crucially, underwent scheduled inpatient intensive decoppering every 2–3 years. She completed her last intensive decoppering course approximately one year before conception. She then voluntarily discontinued all oral maintenance chelators (penicillamine and zinc sulfate) throughout the preconception period and gestation, resulting in a treatment-free interval of over 21 months by the time of delivery. Preconception baseline laboratory evaluations revealed preserved renal compensatory function, with serum creatinine of 85 μmol/L, estimated glomerular filtration rate (eGFR) of approximately 85 mL/min, and baseline 24-hour urinary protein of 0.25 g (1+). Her platelet count had been long-term stable at approximately 140 × 10^9^/L. Her pre-pregnancy body mass index (BMI) was 23.71 kg/m^2^, within the normal range.

#### 2.1.1 Family history

The patient's father and brother were asymptomatic carriers of pathogenic ATP7B variants, consistent with an autosomal recessive inheritance pattern. Her mother was healthy.

#### 2.1.2 Reproductive history

The patient had a first-trimester miscarriage in 2013, a medication-induced abortion in 2014, and a termination of pregnancy at 16 weeks' gestation due to drug exposure in 2020.

### 2.2 Antenatal course

The patient conceived naturally with an estimated delivery date of January 14, 2026. Threatened miscarriage at 6 weeks resolved with progesterone. Intermittent generalized tremors emerged and worsened as gestation advanced. Subclinical hypothyroidism was diagnosed at 7 weeks. Liver function remained stable in the second trimester, with normal transaminases (ALT 12.3 U/L, AST 18.5 U/L) and bilirubin (total 13.7 μmol/L, direct 5.1 μmol/L); albumin was mildly reduced (32.0 g/L) and total protein was 56.4 g/L.

Amniocentesis at 20 + 1 weeks detected a heterozygous ATP7B variant (c.2333G > T, p.Arg778Leu). The variant was classified as pathogenic based on the ClinVar database and confirmed by our laboratory's internal report, which followed the ACMG/AMP guidelines for variant interpretation. Gestational diabetes diagnosed at 26 + 3 weeks was diet-controlled. Second-trimester fetal ultrasound and echocardiography were normal.

No serum ceruloplasmin or 24-hour urinary copper was tested during pregnancy. Liver function declined in the third trimester (albumin fell from 32.0 to 27.0 g/L; total protein from 56.4 to 53.3 g/L). New-onset hypertension (BP 141/92 mmHg) was noted at 38⁺5weeks.

### 2.3 Admission and preoperative Status

The patient was admitted at 38 + 5 weeks of gestation due to new-onset hypertension.

#### 2.3.1 General and neurological examination

BP 141/92 mmHg, HR 96 bpm, choreiform gait, dysarthria, bilateral grade 4/5 limb weakness, positive Babinski signs, and cerebellar ataxia. K-F rings had been documented at diagnosis; slit-lamp examination was not repeated on admission.

#### 2.3.2 Obstetric examination

Fundal height 35 cm, LOA presentation, FHR 150 bpm, cervix closed, Bishop score of 2.

#### 2.3.3 Preeclampsia diagnosis

New-onset hypertension and proteinuria (24-hour urinary protein, 0.609 g) met the diagnostic criteria for preeclampsia, without severe features.

#### 2.3.4 Auxiliary examinations

Abdominal ultrasound revealed a heterogeneous hepatic echotexture without ascites, splenomegaly (30.0 mm), or portal dilatation, indicating compensated cirrhosis. Gallbladder wall thickening (5.1 mm) with polyps was also noted (Figure 1). Obstetric ultrasound confirmed a singleton fetus (EFW 3,400 g, LOA) with a single nuchal cord and normal amniotic fluid volume.

**Figure 1:**
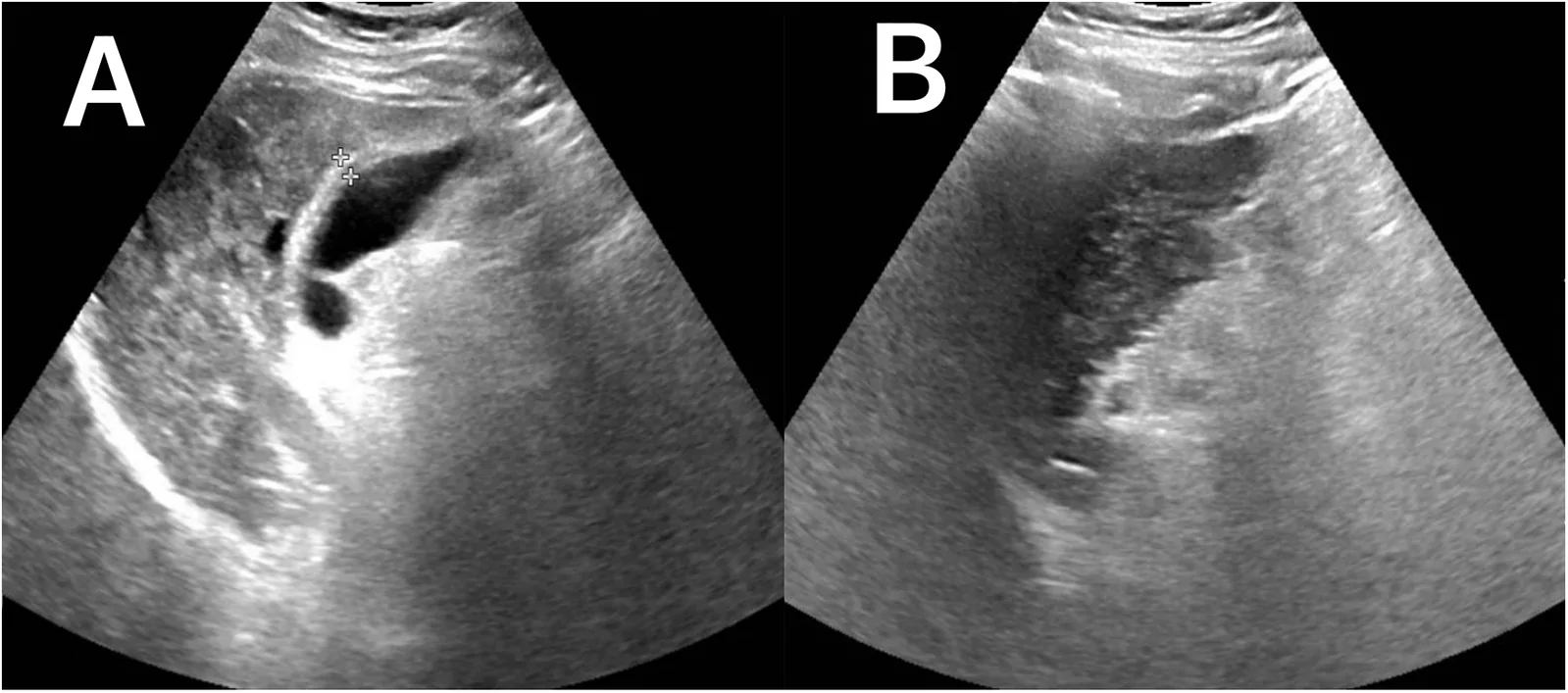
Abdominal ultrasound on admission. **(A)** Hepatic parenchyma with heterogeneous echotexture and no definite cirrhotic nodules or regenerative macronodules, suggesting compensated cirrhosis without overt nodular transformation. **(B)** Gallbladder wall thickening (5.1 mm) with an intraluminal polypoid lesion (approximately 4 mm), consistent with chronic hepatobiliary involvement secondary to long-standing Wilson's disease. No gallstones, pericholecystic fluid, or sonographic Murphy's sign was observed.

#### 2.3.5 Laboratory findings

Liver function tests showed preserved hepatocellular integrity despite reduced synthetic function, as indicated by the following: total protein 53.3 g/L, albumin 27.0 g/L, ALT 15.0 U/L, AST 25.0 U/L, total bilirubin 14.1 μmol/L (conjugated 0 μmol/L, unconjugated 7.8 μmol/L). Transaminase and bilirubin levels remained normal, whereas hypoalbuminemia reflected reduced synthetic reserve. PT 13.8 s, PTA 70.5%, APTT 28.6 s, TT 17.0 s, FIB 2.85 g/L, and platelet count 141 × 10⁹/L. Elevated levels of FDP (13.43 μg/mL) and D-dimer (5.08 mg/L) were consistent with pregnancy-related hypercoagulability and preeclampsia-associated microvascular activation, rather than hepatic failure. Serum ceruloplasmin was 81 mg/L, and urinalysis showed 2 + proteinuria (24-hour urine protein, 0.609 g). The Child-Pugh score was 6, which was consistent with compensated cirrhosis (Class A).

### 2.4 Diagnostic scoring for wilson's disease

We applied the Leipzig scoring system to clinical and laboratory data obtained at admission for delivery (January 2026). Although 24-hour urinary copper was not measured due to the urgent management of preeclampsia, the patient presented with neurological involvement (score 1), had a serum ceruloplasmin level of 81 mg/L (score 2), and a documented history of positive K-F rings since 2003 (score 1). The cumulative score of 4 confirmed the diagnosis, which had already been established for 23 years. Postpartum reassessment (March 2026) confirmed marked cupruria (1,295.72 μg/24 h), which further supported the diagnosis. Detailed scoring is presented in Table 1.

**Table 1:** Leipzig score for wilson's disease (at delivery admission, January 2026).

Diagnostic Category	At Delivery Admission	Score
Neurological Symptoms	Mild (tremor, ataxia, dysarthria)	1
Serum Ceruloplasmin	81 mg/L (< 100 mg/L)	2
24-hour Urinary Copper	Not measured	0
Kayser-Fleischer (K-F) Rings	Not examined on admission (positive historically),	1
Hepatic Copper Content	Not measured	0
ATP7B Gene Mutation	Data unavailable (previously identified heterozygous variant)	0
Total Score		4

Not collected due to urgent preeclampsia workup and patient compliance issues. Documented at initial diagnosis in 2003; slit-lamp not repeated on admission.

### 2.5 Management and delivery

Urgent MDT consultation was scheduled. The decision to proceed with emergency cesarean delivery was multifactorial rather than based on any single indication. First, the patient had an unfavorable cervix (Bishop score 2), rendering induction of labor unlikely to succeed. Second, her progressive neurological deterioration—worsening tremors, ataxia, and dysarthria—raised concern for potential aspiration and further neurological compromise during prolonged labor. Third, the new-onset preeclampsia with worsening hypoalbuminemia (serum albumin decreasing from 32.0 to 27.0 g/L) suggested a trend toward hepatic decompensation, and the risks of expectant management (eclampsia, placental abruption, or acute liver failure) were considered to outweigh the surgical risks in a patient with compensated cirrhosis. Fourth, at 38 + 5 weeks of gestation, fetal lung maturity was presumed, and the risk of intrauterine demise outweighed the risks of preterm delivery. The combination of these obstetric, neurological, and hepatic factors necessitated expedited cesarean delivery. Neurology recommended continuous monitoring; obstetrics initiated intravenous magnesium sulfate for seizure prophylaxis (4 g loading dose over 20 min, then 1 g/h infusion for 24 h postoperatively), with routine monitoring of deep tendon reflexes and respiratory rate. The anesthesiology team favored spinal neuraxial anesthesia over general anesthesia to reduce hepatic drug load and avoid respiratory depression. Coagulation parameters were within acceptable limits (PT 13.8 s, PTA 70.5%, APTT 28.6 s, platelet count 141 × 10⁹/L). Emergency cesarean delivery was performed under spinal anesthesia, yielding a healthy male neonate (Apgar scores 9, 10, and 10; birth weight 3.42 kg). The patient was discharged on postoperative day 4, with chelation therapy restarted.

### 2.6 Postpartum follow-up

Following discharge, the patient promptly resumed systemic copper chelation therapy as prescribed, including penicillamine and DMPS. The antihypertensive regimen consisted of nifedipine and labetalol. Due to the potential excretion of copper metabolites and chelating agents in breast milk, exclusive formula feeding was strongly recommended and adhered to, in accordance with international guidelines (7, 8), which discourage breastfeeding during active full-dose chelation due to limited safety data on agent excretion in breast milk and the theoretical risk of neonatal copper deficiency. The neonate demonstrated steady weight gain under this nutritional regimen.

The patient was readmitted on March 3, 2026, primarily for medication refill and comprehensive reevaluation. During this admission, slit-lamp re-examination at the specialized hepatology center confirmed persistent bilateral Kayser-Fleischer rings, indicative of ongoing copper overload despite oral chelation. Laboratory tests revealed the 24-hour urinary copper level of 1,295.72 μg and the serum ceruloplasmin level of 39.7 mg/L, which were measured after the patient had resumed systemic chelation therapy and thus primarily represent pharmacologically enhanced cupruria rather than the baseline copper burden present at delivery (Figure 2). Notably, liver function had substantially improved compared with the admission values: total protein 67.7 g/L, albumin 41.2 g/L, ALT 16.1 U/L, AST 19.9 U/L, total bilirubin 11.0 μmol/L, and direct bilirubin 3.3 μmol/L, all within normal ranges. Similarly, FDP and D-dimer had normalized (3.44 μg/mL and 0.62 mg/L, respectively), confirming that the elevated admission values were pregnancy- and preeclampsia-related rather than indicative of hepatic synthetic failure. Stepwise DMPS infusion combined with neuroprotective therapy was administered, resulting in clinical improvement. The patient was discharged on April 23, 2026, in stable clinical condition and with confirmed medication compliance.

**Figure 2:**
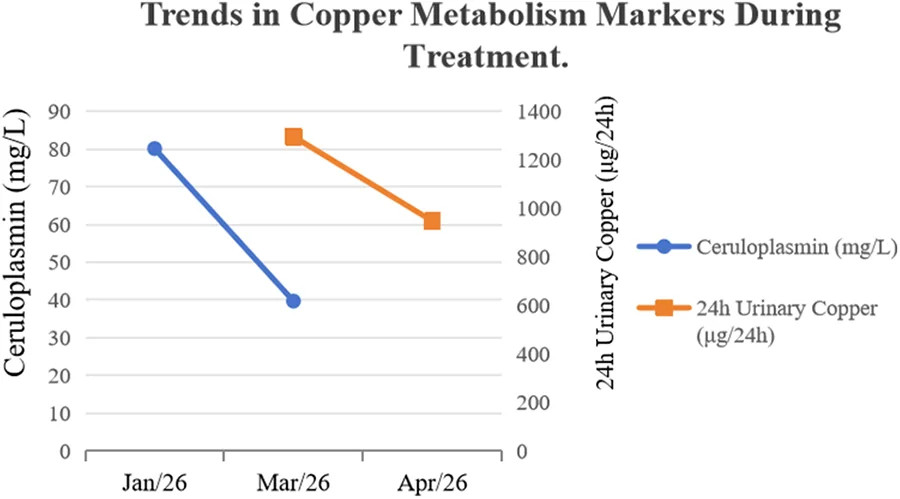
Dynamic changes of laboratory indices during the postpartum follow-up. The blue line represents serum ceruloplasmin levels, and the orange line represents 24 h urinary copper excretion. Both markers showed a significant decrease following treatment initiation.

The patient's full chronological clinical course is summarized in Table 2.

**Table 2:** Chronological summary of clinical events, laboratory examinations and genetic findings in the patient.

Timeline	Key Clinical Events	Key Investigations & Condition
2003	Diagnosis of hepatolenticular WD; Nodular cirrhosis	Positive K-F rings; Abnormal copper metabolism
2006 (Pre-pregnancy)	Chronic renal impairment	Persistent proteinuria (+)
2024	Pre-conception preparation; Self-discontinuation of all chelating medications	Asymptomatic interval
2025-04-09	Last Menstrual Period (LMP); Natural conception	Unremarkable early pregnancy
2025-05-28 (7 Gestational Weeks)	Exacerbated paroxysmal systemic tremors;	no decoppering therapy administered
2025-08-28 (20 + 1 Gestational Weeks)	Amniocentesis for prenatal genetic testing	*ATP7B* mutation: c.2333G > T (heterozygous pathogenic variant)
2026-01-05 (38 + 5 Gestational Weeks)	Outpatient BP 141/92 mmHg; preeclampsia diagnosed	Urinary protein 2+, 24-hour urinary protein 0.609 g
2026-01-06	Cesarean delivery	neonate 3.42 kg, Apgar score 9-10-10
2026-03-03	Readmission for re-evaluation + Chelation therapy	Urine protein negative; 24 h urinary copper 1,295.72 μg/24 h
2026-04-23	Discharged with improved copper chelation status	Stable condition
2026-06	Postpartum follow-up; Continuation of chelation therapy	No specific investigations performed; Stable clinical condition; Medication compliance confirmed

WD, Wilson's disease; K-F rings, Kayser–Fleischer rings; LMP, last menstrual period; BP, blood pressure.

## 3 Discussion

### 3.1 Wilson disease and pregnancy: guidelines and the paradox of outcome

The central paradox of this case is that a WD patient who discontinued chelation therapy for more than 21 months avoided acute liver failure during pregnancy. We propose a “hepatic functional reserve” hypothesis, suggesting that 21 years of periodic intensive decoppering might have maintained hepatic copper below the decompensation threshold, thereby providing a critical buffer. However, we emphasize that, in our patient, this hypothesis lacks direct histopathological confirmation, quantitative hepatic copper measurement, or elastography, and thus remains an unproven inference rather than a demonstrated mechanism. She remained untreated for more than 21 months periconceptionally without hepatic failure, providing circumstantial support for this hypothesis, albeit unproven.

First, from the standpoint of copper metabolic kinetics and the reversibility of hepatic fibrosis, periodic intensive therapy may delay the exhaustion of hepatic reserve (9–12). In WD, copper accumulation is a slow and progressive process, with the liver serving as the primary site of storage and the most severely affected organ (13). In untreated patients, hepatic copper drives fibrosis and eventual failure (10, 14). DMPS, the chelator used in our patient's scheduled inpatient regimens, has been shown to significantly improve liver stiffness and reduce fibrotic markers (HA, LN, CⅣ, PⅢNP) in WD patients when combined with adjunctive therapy (15, 16). These findings indicate that intensive regimens can reverse or delay fibrosis. In our patient, decoppering every 2–3 years over 21 years likely constituted a “pulse” deep clearance, systematically lowering total body copper burden and maintaining hepatic copper levels below the safety threshold.

Second, hepatic reserve can be objectively quantified. A study of 166 WD patients demonstrated that shear wave elastography (SWE) correlates with the ALBI score, serving as a non-invasive assessment tool for hepatic reserve (16). In our patient, admission ALBI grade was 1 (Child-Pugh A), indicating preserved reserve. The rapid postpartum recovery of albumin (from 27.0 to 41.2 g/L) and total protein (from 53.3 to 67.7 g/L) provides direct evidence of reversible hepatic dysfunction—the hallmark of functional reserve (17).

Third, comparison with published WD pregnancy fatalities highlights protective factors. Shimono et al. reported a WD patient who discontinued penicillamine upon discovering pregnancy, developed hemolytic crisis on the day of delivery, and died of hepatic failure on day 50; autopsy revealed massive necrosis (13). Another case described a WD patient who stopped chelation for only 3 days in the third trimester and progressed to acute liver failure with ascites and coagulopathy (18). The key difference lies in the timing of interruption: in comparator cases, chelation was stopped during pregnancy, when estrogen-driven copper mobilization from tissue stores triggers fulminant failure (4). Our patient discontinued therapy more than 21 months before delivery, avoiding acute gestational copper mobilization. A meta-analysis of 16 WD pregnancy studies reported a 20% spontaneous abortion rate overall, with significantly lower rates in treated patients (OR: 0.47, 95% CI: 35%–63%) (19), reinforcing the protective value of continuous therapy.

Finally, this reserve is finite and exhaustible. Our patient still developed neurological deterioration and preeclampsia, indicating that the reserve was not unlimited (9). In interpreting the postpartum laboratory data cited in this discussion, it is important to note that the markedly elevated 24-hour urinary copper level (1,295.72 μg) was obtained during readmission on March 3, 2026, after chelation therapy had been resumed. Therefore, this value should not be interpreted as direct evidence of the severity of copper overload at the time of delivery, but rather as an indicator of the patient'cupruretic response to resumption of pharmacologic intervention.

This hypothesis should not be interpreted as endorsing treatment interruption, but rather as a possible mechanism for relative tolerance in some patients. It reinforces the key message: even with preserved hepatic reserve, treatment discontinuation causes significant harm, and continuous maintenance therapy with rigorous monitoring remains irreplaceable (7, 8). A widespread misconception among WD patients is that discontinuing chelators reduces teratogenic risk. However, clinical guidelines clearly distinguish between low-dose maintenance decoppering and high-dose exposure to chelating agents (7, 8). The minor theoretical fetal risks associated with dose-adjusted standard chelation are far outweighed by life-threatening maternal complications arising from long-term untreated copper overload (1, 8). The timing of delivery in this case was determined primarily to reverse progressive maternal organ damage rather than to avoid fetal drug exposure, a decision that reflects the core principle of balancing maternal and fetal safety.

This section critiques the combined chelation therapy approach. The EASL (2012) and AASLD (2023) guidelines explicitly recommend (i) preconception multidisciplinary planning; (ii) continuous chelation throughout pregnancy, with dose reduction in the third trimester (typically 25%–50%) rather than complete cessation; and (iii) quarterly monitoring of 24-hour urinary copper and liver function (3, 7). Complete chelation withdrawal for more than 21 months, with no monitoring whatsoever, constitutes an indefensible deviation from these guidelines and warrants unequivocal condemnation. The preconception regimen (penicillamine, DMPS, and zinc) is not endorsed as first-line maintenance by the EASL/AASLD guidelines (3, 7) and is generally reserved for refractory cases owing to its potential for increased toxicity. Moreover, in the setting of multi-agent therapy, the standardized 24-hour urinary copper target (200–500 μg/24 h) established for single-agent maintenance becomes unreliable, because DMPS and penicillamine alter copper excretion kinetics unpredictably, thereby precluding accurate assessment of chelation efficacy or over-chelation risk. In a prospective study, Iorio et al. (20) demonstrated that female WD patients receiving long-term adherent maintenance therapy retained normal ovarian reserve (AMH and AFC) and fertility potential comparable to those of healthy controls. In their cohort, reproductive impairment and disease progression occurred almost exclusively among nonadherent patients. These findings reinforce that strict adherence to therapy—rather than fortuitous outcomes following treatment suspension—is the cornerstone of safe reproductive outcomes in WD patients of childbearing age.

### 3.2 Maternal hepatic and neurological considerations during pregnancy

Pregnancy induces physiological changes that affect WD: estrogen-induced hepatic copper mobilization may elevate transaminases, and hemodilution lowers serum ceruloplasmin, rendering standard laboratory monitoring less reliable. On admission, our patient demonstrated preserved hepatic synthetic function (PT 13.8 s, platelets 141 × 10^9^/L, no ascites), but hypoalbuminemia (27.0 g/L) indicated reduced hepatic reserve, consistent with Child-Pugh A cirrhosis.

From a hepatological perspective, the primary concern was whether the patient's Child-Pugh A cirrhosis could tolerate surgical stress (3). Preserved coagulation and the absence of ascites were consistent with a compensated state, whereas hypoalbuminemia indicated reduced hepatic reserve. Delivery would eliminate estrogen-driven copper mobilization, thereby providing a window for intensified postpartum chelation (7).

From a neurological perspective, WD patients with neurological involvement are susceptible to exacerbation of tremors and dysarthria following anesthesia, particularly with sedative-hypnotics (21). The team recommended avoiding agents with prolonged central nervous system (CNS) effects. Our patient developed neurological deterioration during pregnancy—worsening tremors, ataxia, and dysarthria—which was attributed to copper mobilization in the absence of chelation therapy. This deterioration, combined with hepatic concerns, informed the decision to expedite delivery. The neurological impairment that emerged in the first trimester and worsened with advancing gestation underscores the vulnerability of WD patients with pre-existing neurological involvement to copper dyshomeostasis during pregnancy.

### 3.3 Preeclampsia, timing, and mode of delivery

Obstetric perspective: timing of delivery. The team weighed the risk of progression from preeclampsia (proteinuria 0.609 g/24 h and new-onset hypertension) to eclampsia or placental abruption against the surgical risks in a patient with cirrhosis (4, 22). Notably, the patient's concurrent gestational diabetes mellitus (GDM) may have served as an additional and potentially confounding risk factor for preeclampsia, independent of copper overload. The Bishop score of 2 rendered induction of labor unlikely to succeed. At 38 + 5 weeks of gestation, fetal lung maturity was confirmed, and the risk of intrauterine fetal demise outweighed the risks of preterm delivery, favoring expedited cesarean delivery.

Balancing maternal and fetal risks: Expedited cesarean delivery at 38 + 5 weeks was supported by three converging factors: the patient's worsening preeclampsia and hypoalbuminemia posed imminent risks of eclampsia and hepatic decompensation; fetal pulmonary maturity had been confirmed at term; and delivery would relieve gestational hemodynamic overload and enable intensified chelation in the immediate postpartum period. The decision to proceed with emergency cesarean delivery was multifactorial rather than based on any single indication: (1) an unfavorable cervix (Bishop score 2), which made induction unlikely to succeed; (2) progressive neurological deterioration, which raised concern about aspiration and further neurological compromise during prolonged labor; (3) new-onset preeclampsia and worsening hypoalbuminemia, which suggested a trend toward hepatic decompensation; and (4) confirmed fetal pulmonary maturity at 38 + 5 weeks, at which point the risk of intrauterine fetal demise outweighed the risks of expedited delivery. Collectively, these obstetric, neurological, and hepatic factors supported the decision for expedited cesarean delivery.

Admittedly, this patient did not receive scheduled MDT assessments during antenatal care, a clear deviation from current guidelines (7). Although favorable outcomes were achieved only after urgent MDT consultation at 38 + 5 weeks, this does not justify the gap; rather, it underscores that even delayed MDT intervention can be lifesaving, whereas early and continuous MDT involvement would likely have mitigated the observed progressive neurological and hypertensive complications.

### 3.4 Anesthetic management: neuraxial versus general anesthesia

Anesthetic perspective: neuraxial versus general anesthesia. Spinal anesthesia was selected because it avoids hepatic drug metabolism, preserves airway reflexes (critical given the patient's cerebellar ataxia), enables immediate neurological assessment, and avoids systemic opioid exposure (23, 24). The principal risk was spinal hematoma in the setting of thrombocytopenia; however, the patient's platelet count (141 × 10^9^/L) exceeded the contraindication threshold of < 80 × 10^9^/L.

In contrast, general anesthesia offers better hemodynamic control but raises several concerns: hepatic metabolism of anesthetic agents (barbiturates should be avoided), exacerbation of neurological symptoms from hypnotic-sedatives, difficult airway due to dystonia, and potential hepatotoxicity from halogenated anesthetic agents (21, 25). If general anesthesia is required, atracurium and cisatracurium are the preferred muscle relaxants because they undergo Hofmann elimination, a process independent of hepatic and renal function (26).

### 3.5 Postpartum management: the critical transition

Patients with WD are particularly vulnerable during the postpartum period. Rapid estrogen withdrawal and physiological diuresis can alter copper distribution, potentially precipitating a disease flare. In addition, postpartum fatigue and the demands of infant care frequently contribute to non-adherence to treatment.

Following discharge, the patient promptly resumed systemic copper chelation therapy as prescribed, including penicillamine and DMPS. The antihypertensive regimen consisted of nifedipine and labetalol. Given the limited safety data on the potential excretion of copper metabolites and chelating agents into breast milk, as well as the theoretical risk of neonatal copper deficiency, exclusive formula feeding was strongly recommended and strictly implemented, consistent with international guidelines (3, 7, 8) that discourage breastfeeding during active full-dose chelation. The neonate demonstrated steady weight gain with this feeding regimen.

The patient was readmitted on March 3, 2026, primarily for medication refill and comprehensive reevaluation. During this admission, slit-lamp re-examination at the specialized hepatology center confirmed persistent bilateral Kayser-Fleischer rings, indicating ongoing copper overload despite oral chelation. Laboratory tests revealed a 24-hour urinary copper excretion of 1,295.72 μg and a serum ceruloplasmin level of 39.7 mg/L; because these values were obtained after the patient had resumed systemic chelation therapy, they reflected pharmacologically enhanced cupruria rather than the baseline copper burden at delivery. Notably, liver function had substantially improved compared with admission values: total protein 67.7 g/L, albumin 41.2 g/L, ALT 16.1 U/L, AST 19.9 U/L, total bilirubin 11.0 μmol/L, and direct bilirubin 3.3 μmol/L, all within normal limits. Similarly, FDP and D-dimer had normalized (3.44 μg/mL and 0.62 mg/L, respectively), confirming that the elevated values at admission were related to pregnancy and preeclampsia rather than to hepatic synthetic failure. The patient received a stepwise infusion of DMPS combined with neuroprotective therapy; this regimen resulted in clinical improvement. She was discharged on April 23, 2026, in stable clinical condition and with confirmed adherence to the prescribed medication regimen.

We recommend (i) resuming full chelation therapy within 12 h postpartum (or continuing maintenance chelation in patients already receiving it); (ii) monitoring liver function and urinary copper weekly for the first month and then monthly for three months; (iii) actively screening for postpartum depression, given its higher prevalence in chronic disease populations; and (iv) neonatal evaluation, including measurement of cord blood copper and ceruloplasmin (both of which are physiologically low at birth) and follow-up for neonatal cholestasis, a rare but reported complication.

### 3.6 Implications for future pregnancies and long-term counseling

Women with WD who have had an uneventful pregnancy can generally expect similarly favorable outcomes in subsequent pregnancies, provided disease control is sustained. However, each pregnancy imposes a cumulative oxidative stress on the liver. We recommend that patients undergo a comprehensive preconception assessment—including hepatology evaluation, liver elastography, and 24-hour urinary copper excretion—to establish a new baseline. Genetic counseling regarding autosomal recessive inheritance should also be provided: the recurrence risk is 25% if the partner is a carrier, and prenatal diagnosis via chorionic villus sampling or amniocentesis is available, although it is rarely requested. For long-term contraception, estrogen-containing oral contraceptives are relatively contraindicated because of their cholestatic effects; progestin-only pills, intrauterine devices, or barrier methods are therefore preferred. Finally, patients should be reassured that pregnancy does not accelerate WD progression in those who remain adherent to treatment; rather, it serves as a stress test for hepatic reserve.

In accordance with EASL guidelines (7), we emphasize mandatory preconception MDT evaluation, full ATP7B gene sequencing, monthly copper monitoring during pregnancy, and urgent MDT assessment for new neurological or hypertensive symptoms (3, 7, 8). Combined chelation therapy should be resumed promptly in the postpartum period; formula feeding is recommended, and heterozygous offspring should undergo routine copper screening during childhood (3, 7, 8).

### 3.7 Limitations of this case

This case has several limitations that should be considered when interpreting our findings.

Most importantly, our proposed “hepatic functional reserve” hypothesis remains unsubstantiated, as we did not perform quantitative hepatic copper measurements or elastography either before or during pregnancy. This hypothesis should therefore be considered a *post-hoc* explanatory framework rather than a validated mechanism, and future studies incorporating objective assessments of hepatic reserve are warranted. Additionally, routine gestational copper monitoring (including serial 24-hour urinary copper and free copper measurements) was not performed during the entire pregnancy, precluding precise tracking of copper overload progression. Results of complete maternal ATP7B sequencing were not available in the medical records; the patient was diagnosed clinically based on classic clinical manifestations and Kayser-Fleischer rings; however, the specific biallelic mutations remained undocumented.

Prior to pregnancy, the patient did not undergo upper gastrointestinal endoscopy to rule out esophageal or gastric varices, nor was pre-pregnancy transient elastography performed. Consequently, the precise impact of gestational hemodynamic changes on portal pressure could not be assessed. Furthermore, although prenatal amniocentesis identified the fetus as a heterozygous carrier of the ATP7B variant (c.2,333G > T, p.Arg778Leu), comprehensive neonatal genetic confirmation and detailed biochemical and hepatic function tests were omitted during the early postnatal period due to the infant's asymptomatic status, normal growth parameters, and absence of clinical signs of copper metabolism dysfunction. The absence of systematic neonatal copper monitoring and formal pediatric hepatology follow-up constitutes an additional limitation.

Despite these limitations, this case provides targeted guidance for similar high-risk patients—primigravidae of advanced maternal age with long-term multisystem WD and prolonged preconception chelation interruption. Consistent with EASL guidelines (7), we emphasize mandatory preconception MDT evaluation, full ATP7B sequencing, monthly gestational copper monitoring, and urgent MDT response to new neurological or hypertensive symptoms (7, 8). Postpartum combined chelation should be resumed promptly; formula feeding is recommended; and heterozygous offspring require routine copper screening in childhood (7, 8). Cases of term delivery following long-term suspension of chelation remain rare; this report provides valuable real-world evidence for this challenging population.

## 4 Conclusion

We report a rare case of successful term delivery in a patient with Wilson's disease (WD) following a prolonged period of treatment interruption. This outcome should not be construed as evidence supporting the notion that prolonged treatment interruption is tolerable. It raises the speculative possibility that a long-term history of rigorous, periodic decoppering might confer a “hepatic functional reserve,” although this hypothesis remains entirely unproven. This reserve, if present, may sustain patients through periods of non-adherence, but it is finite. This case underscores that even if such a reserve exists, it does not replace the need for continuous therapy and vigilant monitoring; furthermore, complete chelation cessation without monitoring constitutes a severe guideline violation that must be strictly avoided, especially during the physiological stress of pregnancy.

## Data Availability

The original contributions presented in the study are included in the article/Supplementary Material, further inquiries can be directed to the corresponding author.
